# Elevated CSF outflow resistance associated with impaired lymphatic CSF absorption in a rat model of kaolin-induced communicating hydrocephalus

**DOI:** 10.1186/1743-8454-7-4

**Published:** 2010-02-10

**Authors:** Gurjit Nagra, Mark E Wagshul, Shams Rashid, Jie Li, J Pat McAllister, Miles Johnston

**Affiliations:** 1 Brain Sciences Program, Department of Laboratory Medicine and Pathobiology, Sunnybrook Health Sciences Centre, University of Toronto, Toronto, Ontario, Canada; 2 Department of Radiology, Stony Brook University, Stony Brook, NY, USA; 3 Gruss Magnetic Resonance Research Center, Albert Einstein College of Medicine, Bronx, NY, USA; 4 Department of Neurological Surgery, Wayne State University School of Medicine, University Health Center, St Antoine Detroit, MI, USA; 5 Department of Neurosurgery, Division of Pediatric Neurosurgery, University of Utah School of Medicine, Salt Lake City, UT, USA

## Abstract

**Background:**

We recently reported a lymphatic cerebrospinal fluid (CSF) absorption deficit in a kaolin model of communicating hydrocephalus in rats with ventricular expansion correlating negatively with the magnitude of the impediment to lymphatic function. However, it is possible that CSF drainage was not significantly altered if absorption at other sites compensated for the lymphatic defect. The purpose of this study was to investigate the impact of the lymphatic absorption deficit on global CSF absorption (CSF outflow resistance).

**Methods:**

Kaolin was injected into the basal cisterns of Sprague Dawley rats. The development of hydrocephalus was assessed using magnetic resonance imaging (MRI). In one group of animals at about 3 weeks after injection, the movement of intraventricularly injected iodinated human serum albumin (^125^I-HSA) into the olfactory turbinates provided an estimate of CSF transport through the cribriform plate into nasal lymphatics (n = 18). Control animals received saline in place of kaolin (n = 10). In a second group at about 3.5 weeks after kaolin injection, intraventricular pressure was measured continuously during infusion of saline into the spinal subarachnoid space at various flow rates (n = 9). CSF outflow resistance was calculated as the slope of the steady-state pressure versus flow rate. Control animals for this group either received no injections (intact: n = 11) or received saline in place of kaolin (n = 8).

**Results:**

Compared to saline injected controls, lateral ventricular volume in the kaolin group was significantly greater (0.087 ± 0.013 ml, n = 27 versus 0.015 ± 0.001 ml, n = 17) and lymphatic function was significantly less (2.14 ± 0.72% injected/g, n = 18 versus 6.38 ± 0.60% injected/g, n = 10). Additionally, the CSF outflow resistance was significantly greater in the kaolin group (0.46 ± 0.04 cm H_2_O.μL^-1^.min, n = 9) than in saline injected (0.28 ± 0.03 cm H_2_O.μL^-1^.min, n = 8) or intact animals (0.18 ± 0.03 cm H_2_O.μL^-1^.min, n = 11). There was a significant positive correlation between CSF outflow resistance and ventricular volume.

**Conclusions:**

The data suggest that the impediment to lymphatic CSF absorption in a kaolin-induced model of communicating hydrocephalus has a significant impact on global CSF absorption. A lymphatic CSF absorption deficit would appear to play some role (either direct or indirect) in the pathogenesis of ventriculomegaly.

## Background

Communicating hydrocephalus is considered by many to be due to obstruction to cerebrospinal fluid outflow. Whether this is strictly true or not is an ongoing debate. Nonetheless, most theories of communicating hydrocephalus incorporate some form of primary or secondary CSF absorption deficit in the pathogenic mechanisms since it has been demonstrated that CSF outflow resistance is elevated in human hydrocephalus and in animal models designed to simulate this condition [1-4].

Our group has developed the concept that extracranial lymphatic vessels are a major CSF absorption pathway as opposed to the generally accepted arachnoid villi and granulation pathways. While there are no lymphatic vessels within the parenchyma of the brain, CSF moves from the subarachnoid space (SAS), through the cribriform plate foramina in association with the olfactory nerves and finally is taken up by an extensive network of lymphatic vessels within the olfactory submucosa. Once CSF has entered the absorbing lymphatics, it is conveyed in progressively larger ducts through various lymph nodes and is deposited in the venous system at the base of the neck [5]. This concept is supported by studies in many mammalian species including humans [6-8] and non-human primates [9,10]. Overall, these experiments demonstrated that lymphatics serve the major function of removing CSF from the SAS [11-14].

With this background, we developed a kaolin model of communicating hydrocephalus in adult rats to assess the role of lymphatic function in hydrocephalus development [15] and observed that lymphatic CSF uptake was compromised significantly [16]. Indeed, the greatest degree of ventriculomegaly was associated with the lowest lymphatic CSF absorption levels.

These data suggest that a CSF absorption deficit contributed in some way to the pathogenesis of hydrocephalus. However, in this kaolin-induced hydrocephalus model it is possible that an obstruction to CSF transport at one location (the cribriform plate and lymphatics) is met by enhanced CSF clearance through alternative absorption sites. These could include other lymphatic vessels or the arachnoid granulations. To begin to answer this question, it would be informative to determine if the global CSF outflow resistance is increased in the kaolin hydrocephalus rat model. To address this issue, we report on measurements of global CSF outflow resistance in this model of adult communicating hydrocephalus. These studies show that outflow resistance is elevated significantly and that a global CSF absorption deficit correlates negatively with ventricular volume.

## Methods

### Animals

A total of 56 female Sprague Dawley rats (234-287 g, average 261.9 g) were used for this investigation (purchased from Harlan, Indiana, USA and Charles River, Canada). The animals were fed lab rat chow (LabDiet 5001) until sacrifice.

### Details of collaboration

The injections of kaolin or saline were performed in the laboratories of Dr. McAllister at the Wayne State University School of Medicine and magnetic resonance imaging (MRI) analysis to measure the ventricular volume were performed at Dr. Mark Wagshul's laboratory at Stony Brook University according to the protocol approved by each institutional Animal Investigation Committee. The lymphatic studies were carried out in Dr. Johnston's laboratory at Sunnybrook HSC in Toronto. These experiments were approved by the ethics committee at the Sunnybrook Health Science Centre and conformed to the guidelines set by the Canadian Council on Animal Care and the Animals for Research Act of Ontario.

At approximately 2.5 weeks following kaolin or saline injection, MRI studies were performed to determine ventricle size. Two separate groups of animals were used to study lymphatic CSF absorption and CSF outflow resistance, for which animals were sent to Toronto following MRI measurements at approximately 3 weeks post injection.

### Induction of communicating hydrocephalus

As described in detail previously [17], the rats were anesthetized with a mixture of 1-3% halothane with 40% O_2_, and using aseptic techniques the skin was incised along the ventral midline of the neck. After the soft tissues were reflected to expose the base of the skull, a 30-gauge needle, custom bent to 30°, was inserted into the SAS between the clivus and the C1 vertebra. The needle was advanced 1.5 - 2.0 mm along the inner surface of the cranial cavity, and a 50-μL sterile suspension of 25% kaolin in saline was injected at the rate of about 6 μl/sec. The surgical incision was closed in layers using absorbable suture (Vicryl) and the rats were allowed to recover. Post-operatively, butorphenol was given subcutaneously (0.05-2.0 mg/kg) every 4-8 h as needed to control pain. Animals that experienced breathing difficulty (coughing) or symptoms of life-threatening increases in intracranial pressure post-operatively received mannitol (1.5 g/kg IV). Sham controls were prepared in a similar fashion but received sterile saline in place of kaolin.

### Assessment of ventricular size

MRI was performed at Wayne State University to measure ventricular size *in vivo*. After anesthesia (87/13 mg/kg ketamine/xylazine) the animal was placed into a 4.7T magnet and coronal and sagittal T1- and T2-weighted images were obtained (TE/TR = 20/700 ms and 67/5000 ms, respectively) on 1.0 mm slice thickness. Ventricular volumes (including the lateral and third ventricles) were calculated from T2 images, starting from the center of the cerebral aqueduct up to the anterior-most portion of the lateral ventricles. Details of the analysis method of extracting accurate ventricular volumes can be found in a previous report from our group [16].

### Assessment of lymphatic CSF absorption

The development of the method to assess lymphatic CSF uptake in the rat has been described in a previous publication [15]. The rapid movement of CSF tracers (dyes or radioactive proteins) into the olfactory turbinates supports a role for lymphatics in CSF absorption and provides the basis of the method for assessing the impact of hydrocephalus on lymphatic CSF transport.

Rats injected with either kaolin (n = 18) or saline (n = 10) were anesthetized initially by placement in a custom-built rodent anesthesia chamber using halothane (4-5%) in O_2_. For the experimental procedure they were maintained with 2-2.5% halothane in O_2 _delivered by a nose cone (Rat Anesthesia Mask, KOPF, Model 906, Tujunga, USA). The animals were placed on a heating pad (Fine Science Tools, Vancouver, Canada) and fixed in position in a Small Animal Stereotaxic device (KOPF, Model 900, Tujunga, USA). The skin over the cranium was removed and the junction of the sagittal and coronal sutures identified as the bregma. A small high-speed micro drill with a rounded tip (Fine Science Tools, Vancouver, Canada) was used to grind away the bone to expose the dural membrane.

A 50 μL Hamilton syringe (Fisher Scientific, Toronto, Canada) with a 30-gauge needle was positioned at 0.92 mm posterior and 1.4 mm lateral to the bregma, and 3.3 mm deep to the dural membrane at the left or right hemisphere. The coordinates were noted from the stereotaxic instrument and adjusted according to the reference values from a rat brain atlas [18]. The needle was loaded with ^125^I-human serum albumin (HSA) and the tip lowered into a lateral ventricle (because ventriculomegaly developed bilaterally in all cases, either the right or left ventricles was used randomly). Fifty μl containing 500 μg of ^125^I-HSA (0.93 MBq/ml, 10 mg/ml, Drax Image, Quebec, Canada) was injected over 30 s. The needle was removed after 1-2 min and the entrance hole sealed with bone wax. After 30 min, the animals were sacrificed by injection of 1.0 ml Euthanyl (i.p.). The carcasses were then frozen for at least 24 h in a freezer.

To facilitate the assessment of radioactivity in the olfactory submucosa and to prevent potential post-mortem tracer contamination from the CSF compartment, the turbinates were cut from frozen tissue. The olfactory turbinates were excised with a fine tooth saw as described in detail in our previous paper and assessed for radioactivity [15]. The samples were placed into pre-weighed glass test tubes for weight determination using a Mettler BB2400 balance and were monitored for radioactivity in a multi-channel gamma spectrometer (Compugamma, LKB Wallac, Turku, Finland).

### Outflow Resistance

At about 3.5 weeks post injection, the animals were anesthetized and the skull stabilized in a stereotaxic device as described above. These animals were either injected with kaolin (n = 9), saline (n = 8), or not injected (intact, n = 11). Following a midline incision, the skin was reflected over the cranium and a hole was drilled into the skull for eventual insertion of a brain cannula into a lateral ventricle for CSF pressure measurement (same stereotaxic co-ordinates as radioactive tracer injection). Animals were then taken out of the stereotaxic frame and a tracheotomy was performed. A three-way stopcock supplying 1.5% O_2 _with isofluorane and air exhaust (Bickford Inc., Wallis Centre, New York, USA) was connected to a 14-gauge catheter (Infusion Therapy System Inc., CE0086, Sandy, USA), which was inserted into the trachea tube. The tracheal catheter was secured with Surehold glue (mixture of ethyl cyanoacrylate and polymethylmethacrylate, Surehold, Chicago, USA) and the skin sutured with 4.0 silk.

Access to the SAS for infusion of artificial CSF was achieved with a laminectomy to expose the SAS at the lumbar level. A 24-gauge needle containing a 1.9 cm catheter (Terumo, Surflo I.V. Catheter 2227) was inserted ~0.25 cm into the SAS using a surgical microscope (Carl Zeiss OPMI 1-FC). Once the catheter was in place the needle was removed. When CSF had filled the catheter, it was connected via an adapter (Lot 99575890 Argon Medical Devices, Athens, USA) to a 5 ml syringe containing sterile Ringer's solution mounted in a syringe pump (Stoelting Co., catalogue # 53200, Wood Dale, USA).

At this point, an Alzet rat brain cannula (Direct Corporation, Cupertino, USA) was inserted into the burr hole with the tip positioned in the lateral ventricle. The cannula was secured to the skull with Surehold glue. Intraventricular pressure was monitored with the brain cannula connected to a pressure transducer (Custom CDX3 with stop cock, Lot 99471637, Richmond Hill, Canada). This system was calibrated first using a water reservoir at the beginning of the surgical procedure. The data were recorded using a data acquisition system (Daq Software, A-Tech Instuments, Toronto, Canada).

A stable baseline pressure was recorded for each experiment before the infusion of Ringer's solution into the lumbar SAS. Flow rates were adjusted incrementally (10, 22, 34, 50, 100 and 153 μL/min) and steady-state pressures were established for 3-6 min at each flow rate before increasing the rate to the next level.

### Analysis of Data

Out of the total of 56 animals, we have included data from 19 rats that were published in a previous report [16]. These include measures of lymphatic function and ventricular volumes following kaolin and saline injections that were collected in experiments conducted over a 3-year period. All data are expressed as the mean ± SEM. The data were assessed as suited with the t-test, linear regression, ANOVA, or the Kruskal-Wallis test and as appropriate, post-hoc Bonferroni (SPSS 17).

## Results

### Development of hydrocephalus

**Figure 1:**
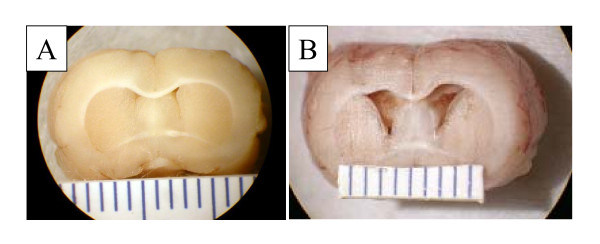
**Example of hydrocephalus induced with administration of kaolin into the basal cisterns**. Note the normal size of the frontal horns of the lateral ventricles in saline injected control animal (A) and the mild enlargement of the lateral ventricles in kaolin injected hydrocephalic animal (B). Scale = 1 mm per line.

**Figure 2:**
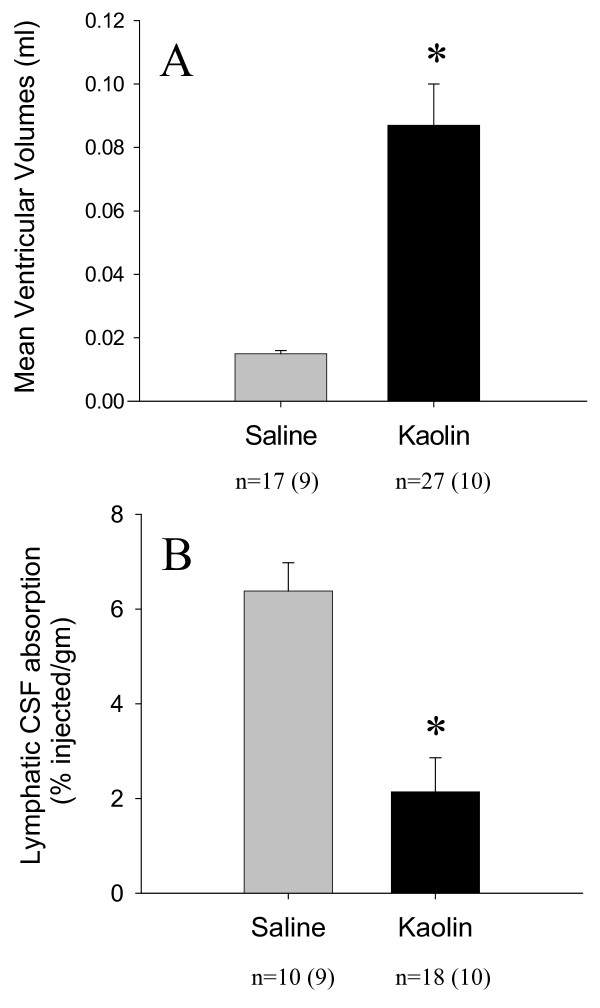
**Ventricle size and lymphatic CSF uptake**. (A) Mean ventricular volumes in the kaolin injected rats were significantly greater (*) than those in the saline injected animals (*p *< 0.0001; independent t-test). (B) The enrichment of radioactive protein tracer in the olfactory turbinates (lymphatic CSF uptake) was significantly less (*) in the animals receiving kaolin (*p *< 0.0001; independent t-test). The numbers of animals in each group are indicated below each histogram. The numbers within the brackets represent data taken from [16].

Details on the morphological changes that accompany this model have been described previously [17]. The cerebral aqueduct is patent in this model and expanded posteriorly. The lack of obstruction in the ventricular system and the patent foramina of Luschka indicated that the hydrocephalus was of the communicating type. Compared to saline-injected controls (a *post-mortem *example is illustrated in Figure 1A), the ventricular system expanded to moderate levels in most kaolin-injected animals (example in Figure 1B). Figure 2A illustrates the average ventricular volumes in the saline and kaolin-injected groups. In the kaolin injected rats the ventricular volumes were significantly greater (0.087 ± 0.013 ml, n = 27) than those observed in the saline injected animals (0.015 ± 0.001 ml, n = 17) (*p *< 0.0001).

### Transport of CSF tracer into olfactory turbinates: comparison between kaolin and saline injected animals

In past studies, we determined that the highest concentrations of ^125^I-HSA were found at 30 min in the middle turbinate area, which represents the bulk of the olfactory turbinates. For this reason, we chose to assess the lymphatic uptake of CSF using tracer enrichment at this location and at that time.

Figure 2B illustrates the average middle turbinate enrichment data assessed at 30 min after tracer injection. Tracer enrichment (*i.e*. movement across the cribriform plate) in the kaolin animals was significantly reduced compared to that observed in saline controls (2.14 ± 0.72% injected/g, n = 18 versus 6.38 ± 0.60% injected/g, n = 10) (*p *< 0.0001). The tracer enrichment in the kaolin animals was only 34% of that observed in the saline controls.

### CSF outflow resistance

**Figure 3:**
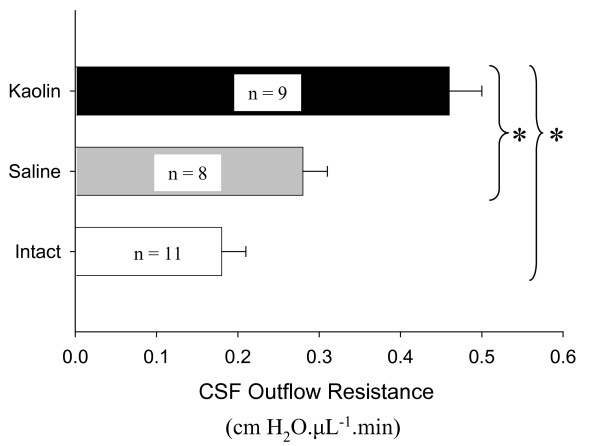
**Average CSF outflow resistance in intact, saline or kaolin-injected animals**. Analysis by both the Kruskal Wallis (*p *< 0.0001) test and ANOVA (*p *< 0.0001) revealed that the 3 groups were significantly different. Post-hoc Bonferroni test indicated that the outflow resistance in the kaolin group was significantly greater than that of the saline (*p *= 0.004) and intact group (*p *< 0.0001) but no significant difference was observed between saline and intact animals.

**Figure 4:**
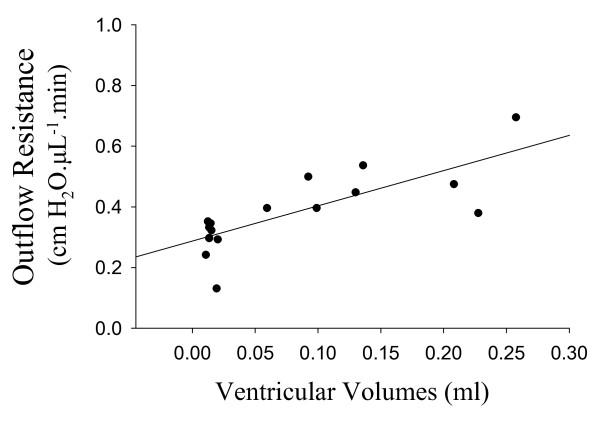
**Relationship between CSF outflow resistance and ventricular volumes measured in the same rats (n = 16)**. These animals include kaolin and saline injected rats used in Figures 2A and 3. No volume measurements were made in intact animals. Please note that we measured CSF outflow resistance in 17 animals. However, the ventricular volume for one of these animals was not available. There was a significant positive correlation between CSF outflow resistance and ventricular volume (*p *= 0.001, Pearson correlation; r^2 ^= 0.583).

The average outflow resistances are illustrated in Figure 3. The CSF outflow resistance for the kaolin group (0.46 ± 0.04 cm H_2_O.μL^-1^.min) was significantly greater than in the saline-injected animals (0.28 ± 0.03 cm H_2_O.μL^-1^.min) *(p *= 0.004) or in the intact, non-injected rats (0.18 ± 0.03 cm H_2_O.μL^-1^.min) (*p *< 0.0001). The difference in outflow resistance between saline and intact controls was not significant (*p *= 0.082). Figure 4 illustrates a significant positive correlation between CSF outflow resistances and ventricular volume (r^2 ^= 0.583, *p *= 0.001).

## Discussion

Our data from this study and from a previous report [16] demonstrate that an absorption deficit occurs at a discrete anatomical location in a kaolin-induced model of communicating hydrocephalus in the rat. At present, we do not know the mechanism by which lymphatic CSF uptake is impaired. It is possible that kaolin induces areas of obstruction to CSF movement within the cranial cavity, possibly due to fibrosis within the SAS. Certainly, collagen deposition has been observed in other models of hydrocephalus. Elevated CSF concentrations of fibroblast growth factor (FGF-2) are associated with hydrocephalus and when FGF-2 is introduced into the ventricular system of rats, CSF formation rate declines and CSF outflow resistance increases [3]. Collagen deposits were observed in the arachnoid membrane and likely impeded CSF flow in this model. However, in these experiments the authors concluded that the ventricles expanded passively due to changes in the subependymal brain parenchyma (an *ex vacuo *type of hydrocephalus).

In any event, a kaolin-induced inflammatory response may generate pro-inflammatory substances that elicit fibrosis in the SAS or in the cribriform plate foramina. If this were the case, CSF movement into extracranial lymphatics might be compromised. However, kaolin deposits were never observed in the area of the olfactory bulb and cribriform plate in gross *post-mortem *examinations of the animals used in this study, and thus we do not believe that kaolin had a direct effect on the foramina of the cribriform plate or on the nasal lymphatics themselves.

The lymphatic CSF absorption deficit appears to correlate with ventricular volumes. However, it is possible that the impediment to lymphatic CSF transport may be a local phenomenon with CSF clearance increasing at other absorption sites to compensate, such that global CSF absorption is relatively unchanged. These sites could include other lymphatic vessels, absorption from the spinal SAS into peri-spinal lymphatics, drainage through the arachnoid projections into the superior sagittal sinus or clearance via the capillary system. If marked compensation were to occur through these other pathways, we would expect that CSF outflow resistance would be close to normal in the kaolin group. However, we observed that outflow resistance was elevated significantly suggesting that the impediment to lymphatic CSF absorption had an impact on global CSF drainage.

This conclusion is supported by several other considerations. First, we know that the movement of CSF through the cribriform plate into nasal lymphatic vessels accounts for at least 50% of global CSF absorption in the rat [19]. Second, in sheep, we also know that about 25% of absorption occurs from the spinal subarachnoid compartment and there is every reason to believe that the majority of this transport is via peri-spinal lymphatic vessels [20]. While not measured in our study, CSF removal from the spinal subarachnoid compartment in the kaolin rat model may have been compromised as well. Third, in sheep in which a similar proportion of CSF absorption occurs through this pathway, sealing the cribriform plate on the extracranial surface leads to reduction in CSF absorption [13,14], elevation of intracranial pressure [21] and a significant increase in CSF outflow resistance [22]. Taken together, the data suggest that the kaolin-induced reduction in lymphatic CSF uptake plays a major role in the increased outflow resistance.

Literature values for CSF outflow resistance measurements in the rat vary considerably. Values from 0.63 to 2.4 cm H_2_O.μL^-1^.min have been reported [23-29] and these are greater than the average value we report in this study (0.18 ± 0.03 cm H_2_O.μL^-1^.min) in intact animals. However, it is difficult to compare values due to methodological differences. Nonetheless, in relative terms, our results agree with previous studies showing elevated outflow resistance with hydrocephalus induction [25-27,29].

### CSF absorption and hydrocephalus

Classically, hydrocephalus has been attributed to an imbalance between CSF production and absorption. Since overproduction of CSF is relatively rare, most investigators have focused on CSF malabsorption as the most likely cause of hydrocephalus. It is hard to ignore this possibility since CSF outflow resistance is elevated in most human [30] or experimental cases [31]. Either CSF movement is restricted within the brain (non-communicating or intraventricular type) or the defect resides at the site of absorption (communicating type or extraventricular type).

In the study reported here, we observed a decline in global CSF absorption in a communicating form of hydrocephalus and this impediment correlated significantly with increasing ventricular size. This would tend to suggest a link between the CSF absorption deficit and hydrocephalus. It is not apparent however, how disruption of CSF drainage through the cribriform plate would lead to a transmantle pressure gradient favoring enlargement of the ventricles since CSF pressure in communicating hydrocephalus would likely rise equally in all intracranial CSF spaces. Indeed, the question of pressure gradients is a perplexing one. Some investigators have failed to measure transmantle gradients in various types of hydrocephalus or have measured gradients that are very small [32-35].

One group has postulated that redistribution of pulsatility within the cranium can lead to ventriculomegaly [36]; the potential effect of impaired lymphatic CSF uptake on compliance could thus lead to redistribution of pulsatility and the ensuing ventricular dilation. Of course, it is important to remember that there are other conditions in which CSF outflow resistance is elevated without hydrocephalus developing such as pseudotumor cerebri, [37].

It seems unlikely therefore, that the pathogenesis of ventriculomegaly can be reduced to a simple 'plumbing problem'. Alternatively, anomalies related to CSF clearance may be 'co-conspirators' in hydrocephalus development but by themselves, not represent the initiating cause of the disorder. We are currently leaning towards the development of a 'two-hit' hypothesis for communicating hydrocephalus in which the CSF absorption deficit is secondary to some primary insult. In this regard, we have evidence that the interstitium of the brain can play a dynamic role in controlling tissue pressure. Modulation of beta-1 integrin-matrix interactions in the periventricular parenchyma reduces interstitial fluid pressure relative to that in the ventricular system. This causes an intra-mantle or intra-parenchymal pressure gradient that favours ventricular expansion [38]. It is possible that pathological disturbances that interfere with integrin-matrix interactions might also impact CSF clearance.

Inflammation is known to affect beta-1 integrin-matrix interactions and induce the interstitial pressure lowering effects in skin [39,40]. In addition, inflammatory cytokines are also known to impair lymphatic pumping activity [41]. Kaolin clearly induces local inflammatory responses in the subarachnoid spaces, such as arachnoiditis, which contribute to the mechanical obstruction seen in models of both obstructive [42] and communicating hydrocephalus [43]. Thus, one could speculate that kaolin first induces matrix changes that lead to intra-mantle pressure gradients favouring ventriculomegaly. However, the recent studies of gene expression changes following kaolin induction in juvenile rats [43] and H-Tx rats with congenital neonatal hydrocephalus [44], as well as immunohistochemical analyses [45], suggest that neuro-inflammation is largely a secondary response to ventriculomegaly. Nevertheless, the inflammatory changes that begin very soon after ventricular expansion could easily exacerbate alterations in integrin receptors and thus intensify the magnitude of the pressure gradients responsible for ventricular expansion, in addition to altering downstream lymphatic function.

In this regard, tracers injected into brain parenchymal tissues convect into the SAS or ventricular system via the Virchow-Robin or perivascular spaces and can be recovered in cervical lymph [46]. This indicates a link between the extracranial lymphatic system and the interstitium of the CNS and implies that events in the brain tissues may have an impact on downstream lymphatic function and possibly lymphatic dysfunction can in turn affect parenchymal interstitial events. Complicating this issue however, is the supposition that parenchymal water may be absorbed into the capillaries of the brain [35]. If this were true, then interstitial fluid would have two possible escape routes, the intracranial vascular system and extracranial lymphatic vessels. How these two systems might interact in the context of the kaolin hydrocephalus model is unknown.

## Conclusions

The data in this report suggest a relationship between CSF outflow resistance and ventricle size in a kaolin-induced hydrocephalus model in rats. The elevation in CSF outflow resistance appears to relate at least in part, to an impediment to CSF clearance through the cribriform plate into extracranial lymphatic vessels. Whether the association between CSF absorption and ventricle size originates from a direct cause and effect relationship or rather arises secondarily from a complex interplay of other unknown physiological parameters, requires further investigation.

## Competing interests

The authors declare that they have no competing interests.

## Authors' contributions

GN: performed the CSF outflow resistance studies and assessed lymphatic CSF uptake; Helped to plan the study and participated in its design and coordination. MEW: conceived of the study, and participated in its design and coordination; Quantified ventricular volumes. JL: injected the rats with kaolin or saline. SR: Quantified ventricular volumes. JPM: conceived of the study, and participated in its design and coordination. MJ: conceived of the study, and participated in its design and coordination.

All authors have read and approved the final version of the manuscript.
